# Joint Lung CT Image Segmentation: A Hierarchical Bayesian Approach

**DOI:** 10.1371/journal.pone.0162211

**Published:** 2016-09-09

**Authors:** Wenjun Cheng, Luyao Ma, Tiejun Yang, Jiali Liang, Yan Zhang

**Affiliations:** 1 Medical Imaging Department, Taizhou Municipal Hospital, No.381 East Zhongshan Road, Taizhou 318000, China; 2 College of Optical and Electronic Technology, China Jiliang University, Xueyuan Street 258, Hangzhou 310018, China; Xuanwu Hospital, Capital Medical Universty, CHINA

## Abstract

Accurate lung CT image segmentation is of great clinical value, especially when it comes to delineate pathological regions including lung tumor. In this paper, we present a novel framework that jointly segments multiple lung computed tomography (CT) images via hierarchical Dirichlet process (HDP). In specifics, based on the assumption that lung CT images from different patients share similar image structure (organ sets and relative positioning), we derive a mathematical model to segment them simultaneously so that shared information across patients could be utilized to regularize each individual segmentation. Moreover, compared to many conventional models, the algorithm requires little manual involvement due to the nonparametric nature of Dirichlet process (DP). We validated proposed model upon clinical data consisting of healthy and abnormal (lung cancer) patients. We demonstrate that, because of the joint segmentation fashion, more accurate and consistent segmentations could be obtained.

## Introduction

Pulmonary disease is a major health problem across the world. It has been reported by world health organization (WHO) that lung cancer causes more than 1.3 million deaths each year [1]. Modern imaging modalities such as computed tomography (CT) and magnetic imaging resonance (MRI) serve as an efficient approach to provide visual examinations for diagnosis and treatment. The development of positron emission tomography (PET) and multi-modality instrument also provides valuable functional information for describing pulmonary conditions. For example, information contained in high resolution lung (CT) images is of great interest for both research and clinical purposes. Given the CT scans of the patient, we can extract region of interest (ROI) from the image like lung field, lung tumor and vertebral column so as to perform subsequent quantitative analysis based on it. For dynamic imaging, we can extract the ROI in each frame and further analyze its motion mechanics from the image domain to evaluate its functionality [2]. In most cases, we need image segmentation as an introductory step to accurately delineate the boundary of lung and other tissues, especially for diagnosis and treatment of cancer. Because in clinical practice we need to estimate the tumor size, evaluate its growth rate and other aspects, which certainly need accurate measurements of tumor boundaries.

Another important application of lung CT is radiation therapy, where shaped radiation beams are aimed from several angles of exposure to intersect at the tumor. As a result, precise treatment intent depends heavily on the segmentation protocols to compute the tumor size and location. However, in current clinical practice, such procedures still rely heavily on laborious tracing by physicians. But small lesions could be unidentifiable for visual inspections. The slight difference of contrast intensity between two types of tissues is also a great challenge for human eyes. So automatic lung field segmentation that can trace the boundary is of great desire. However, accurate and robust algorithms for the segmentation of lungs are still challenging because of the large shape variations for different patients among the population and the ambiguous boundaries due to side effect and complicated structures of lung anatomy.

Previous studies on this topic are mostly devoted to applying models from the computer vision community to medical images of CT, MRI and PET. Representative work includes kernel mean shift [3], region growing [4], adaptive border marching [5] and random walk [6]. In [7], Farahani first extracted features including roundness, circularity from CT images. Then he used ensemble learning to classify each pixel into each organ category by combining multiple classifiers like k-nearest-neighbors, support vector machine and neural network [7]. Based on the fact that lung CT blue scans from different patients share similar boundary structures. The active shape model first learns a template from lung image database via principal component analysis (PCA) and then adapts the template to target image [8, 9]. Further developments on this line of research include combining shape and appearance models while, in the mean time, increasing its robustness to outliers.

Yongqiang managed to combine watershed, active contours, and Markov random field together to extract lung lesions from CT scans [10]. However, their active shape and appearance model can only segment one single object once at a time. Moreover, for small lesion regions like tumors, it is generally difficult to gather enough training examples, in which case, it would be difficult for the trained model to generalize to the objects of small tumors. Besides, most segmentation algorithms need pre-defined number of the overall objects, which is often set in an *ad-hot* manner. While inappropriate setting of this number could lead to either under- and over-segmentation.

As a nonparametric Bayesian model, Dirichlet process (DP) was originally applied to data clustering in the machine learning community [11]. The model is able to automatically infer the number of clusters from the data itself. Since then, several work tried to apply this model to image segmentation by incorporating Markov random fileds or using its dependent counterpart [12] [13]. To enforce cluster sharing among DPs for the whole dataset, Yee Whye proposed the hierarchical Dirichlet process model (HDP) to cluster multiple groups of dataset [14]. In other words, there is common category for the entire dataset and the clusters for each group forms a specific subset of the shared category. The concept of category sharing is also be exploited in medical imaging problems. For example, Jbabdi used the HDP model in connectivity-based parcellation for multiple subjects [13]. Wang derived an hierarchical model for tractography clustering of human brain [15].

In this paper, we propose to utilize the concept of category sharing in lung CT based on the assumption that different lung images are composed by a common set of objects with similar positioning. Thus we can jointly segment multiple lung CT images via hierarchical Bayesian model. The nonparametric DP model is also able to infer new cluster for irregular regions in each image. Moreover, the Dirichlet process (DP) that we construct for segmenting each image is regularized by Markov random field so as to enforce label proximity for neighboring pixels as [12]. Then we construct the hierarchical model to link each image-specific DP. At last, we validate this algorithm on clinical data obtained from both healthy and cancer patients.

The proposed model has three contributions to the clinical practice in lung CT scans:

- We employ the Dirichlet process as the basic building block for image segmentation. Due to its nonparametric nature, it automatically infers the anatomy structure, i.e., number of organs, from the image data. While most current algorithms rely on the pre-defined number of ROIs or manual set seeds for the segmentation algorithm to work. So it requires minimum interventions from physicians.
- We address the problem of shape variations and irregularity by explicitly combining multiple image scans from the population and jointly segmenting them via the hierarchical Bayesian paradigm. Specifically, we assume that lung anatomies in the population origin from the same category of organ and tissue types. But the specific anatomical structure of each image is described by the corresponding Dirichlet process that is regularized by Markov random filed.
- We collected real CT scan data from both healthy patients and abnormal patients with lung tumors. We also provide a performance comparison with the conventional DP segmentation model and random walk that is adopted routinely in medical image segmentation software. The ground truth for segmentation is obtained by a professional physician.

## Methods

### Dirichlet Process

In this section, we follow the terminology from [12] for briefly reviewing Dirichlet process (DP) model. The Dirichlet process *DP*(*α*_0_, *G*_0_) can be conceived as a random distribution over random distributions. It has two parameters, scaling factor *α*_0_(*α*_0_ > 0) and base measure *G*_0_. A random probability measure *G* over measurable space (Θ,A) is sampled from *DP*(*α*_0_,*G*_0_), such that, for any finite partition (*A*_1_, …, *A*_*r*_) of Θ, the random vector (*G*(*A*_1_), …, *G*(*A*_*r*_)) is distributed according to the Dirichlet distribution:

$$\left(G\left(A_{1}\right),\ldots ,G\left(A_{r}\right)\right)∼\text{Dir}\left(\alpha_{0}G_{0}\left(A_{1}\right),\ldots ,\alpha_{0}G_{0}\left(A_{r}\right)\right)$$ (1)

The existence of Dirichlet process was proved by Ferguson [16]. DP has two nice properties

- *Posterior estimation*. Suppose we have observed samples *θ*_1_, …, *θ*_*n*_ from the distribution *G* drawn from DP. The empirical distribution for the observed data points are
  $${\hat{G}}_{n}\left(\theta \right)=\frac{1}{n}\sum \delta_{\theta_{i}}\left(\theta \right)$$ (2)
  where $\delta_{\theta_{i}}$ is a point mass. Then the posterior of *G* is still a DP, $G|\theta_{1},\ldots ,\theta_{n}∼DP\left(\alpha_{0},G_{0}+n{\hat{G}}_{n}\right)$
- *posterior DP*. After observing *θ*_1_, …, *θ*_*n*_, the posterior DP is
  $$G|\theta_{1},\ldots ,\theta_{n}∼DP\left(\alpha_{0}+n,\frac{\alpha_{0}}{\alpha_{0}+n}G_{0}+\frac{n}{\alpha_{0}+n}{\hat{G}}_{n}\right)$$ (3)

As we can see, the new point generated from *G* is either assigned a new value or repeated with previous points (*θ*_1_, …, *θ*_*n*_). The unique values of *θ*_1_, …, *θ*_*n*_ also induce a partitioning of the set [*n*] = {1, …, *θ*_*n*_} into clusters such that within each cluster, *θ* takes on the same value. As a result, we can model a set of data points {*x*_1_, …, *x*_*n*_} with latent parameters {*θ*_1_, …, *θ*_*n*_}, where each *θ*_*i*_ is sampled from *G*. Therefore the latent parameter can be used for data clustering as follows: each data point *x*_*i*_ is determined by *θ*_*i*_ via a parametric function *F*(*θ*_*i*_)

$$\begin{array}{cc} x_{i} & |\theta_{i}∼F\left(\theta_{i}\right)\\ \theta_{i} & |G∼G\\ G & |\alpha_{0},G_{0}∼DP\left(\alpha_{0},G_{0}\right) \end{array}$$ (4)

Because the measure *G* that is sampled from a Dirichlet process is discrete with probability 1, *θ* can take repeated values. So we can model the data points within the same cluster with the same latent parameter *θ*. As we said previously, following the random measure *G*, *θ*_*n*+1_ can either take value from existing set of *θ*_*i*_ or be assigned a new value with probability of $\frac{\alpha_{0}}{\alpha_{0}+n}$.

### Dirichlet Process for image segmentation

Image segmentation amounts to assigning each image pixel or super-pixel a label based on its extracted features (SIFT, HOG). From the perspective of data clustering, it is a process that groups pixels into several clusters. Orbanz proposed to regularize the Dirichlet process with Markov random field so that spatial information could be incorporated for segmentation [12].

Markov random field models the distribution of random variables over an undirected graph. Formally, a graph G=(V,E) is a finite collection of nodes $V=\theta_{1},\ldots ,\theta_{n}$ and set of edges E. The random variable at a node is only dependent on the random variables in its Markov blanket *B*_*i*_. The Hammmersley-Clifford theorem gives its general form as

$$P\left(\theta \right)=\prod_{c\in C}F_{c}\left(\theta \right)$$ (5)

where C is the set of maximal cliques of G. *P*(*θ*) can also be expressed by energy function

$$P\left(\theta \right)=\frac{1}{Z_{H}}\exp\left(-H\left(\theta_{1},\ldots ,\theta_{n}\right)\right)$$ (6)

where *Z*_*H*_ = ∑_*θ*_ exp(−*H*(*θ*_1_, …, *θ*_*n*_)) is the partition function to ensure that the distribution is normalized. Furthermore, the distribution *P*(*θ*) can be decomposed into a node-wise term *E*(*θ*) and an interaction term *M*(*θ*).

$$\begin{array}{c} P\left(\theta \right)\propto E\left(\theta_{1},\ldots ,\theta_{n}\right)M\left(\theta_{1},\ldots ,\theta_{n}\right)\\ E\left(\theta_{1},\ldots ,\theta_{n}\right)=\frac{1}{Z_{E}}\exp\left(-\sum_{i}H_{i}\left(\theta_{i}\right)\right)\\ M\left(\theta_{1},\ldots ,\theta_{n}\right)=\frac{1}{Z_{M}}\exp\left(-\sum_{c\in C}H_{c}\left(\theta_{c}\right)\right) \end{array}$$ (7)

As in [12], we can model the node-wise term with the measure sampled from a DP, while the interaction energy function can be expressed by binary similarity, i.e., *H*_*c*_(*θ*)*i*|*θ*_−*i*_) = *λ*∑_*l*∈∂(*i*)_ *w*_*il*_ *δ*_*S*_*i*_,*S*_*l*__. *w*_*il*_ is the weight of the edge between node *i* and node *l*, *δ*_*S*_*i*_,*S*_*l*__ is the Kronecker symbol.

The resulting model can be formulated as

$$\begin{array}{cc} \left(x_{1},\ldots ,x_{n}\right) & ∼\prod_{i=1}^{n}F\left(x_{i}|\theta_{i}\right)\\ \left(\theta_{1},\ldots ,\theta_{n}\right) & ∼M\left(\theta_{1},\ldots ,\theta_{n}\right)\prod_{i=1}^{n}G\left(\theta_{i}\right)\\ G & ∼DP(\alpha_{0},G_{0}) \end{array}$$ (8)

### Hierarchical Dirichlet Process

For each lung CT image, we can construct a regularized DP for segmentation. However, those CT images from different slice positions of the same patient or similar slice positions of different patients, could share the same set of object category (i.e., cluster labels). So if we link each image-specific DP together, more consistent results might be expected. Formally, we can construct a hierarchical Dirichlet process that defines a distribution over a set of random measures *G*_*i*_ with a global base measure *G*_0_ over measurable space (Θ,A). The global base measure is sampled from a DP with scaling parameter *γ* and base measure *H*

$$G_{0}|\gamma ,H∼DP\left(\gamma ,H\right)$$ (9)

The random measure for each group are conditional independent given *G*_0_, in that each *G*_*i*_ is a draw from a DP with *G*_0_ as base measure

$$G_{i}|\alpha_{0},G_{0}∼DP\left(\alpha_{0},g_{0}\right)$$ (10)

The overall generative model for this hierarchical DP model can be expressed

$$\begin{array}{cc} x_{ji}|\theta_{ji} & ∼F(\theta_{ji})\\ \theta_{ji}|G_{j} & ∼G_{j}\\ G_{i}|\alpha_{0},G_{0} & ∼DP(\alpha_{0},g_{0})\\ G_{0}|\gamma ,H & ∼DP(\gamma ,H) \end{array}$$ (11)

where *x*_*ji*_ denotes the feature for *j*-th pixel in the *i*-th image. We can still incorporate the Markov random field model into each sub-DP model.

$$\begin{array}{cc} \left(x_{i1},\ldots ,x_{in}\right) & ∼\prod_{i=1}^{n}F\left(x_{ij}|\theta_{ij}\right)\\ \left(\theta_{i1},\ldots ,\theta_{in}\right) & ∼M\left(\theta_{i1},\ldots ,\theta_{in}\right)\prod_{i=1}^{n}G_{i}\left(\theta_{j}\right)\\ G_{i}|\alpha_{0},G_{0} & ∼DP(\alpha_{0},g_{0}) \end{array}$$ (12)

Where *G*_0_ is the global base measure generated the DP model in the top. Inference of this resulting model can be done by Gibbs sampling based on the Chinese franchise [14] and the expectation-maximization like algorithm.

## Results

**Fig 1:**
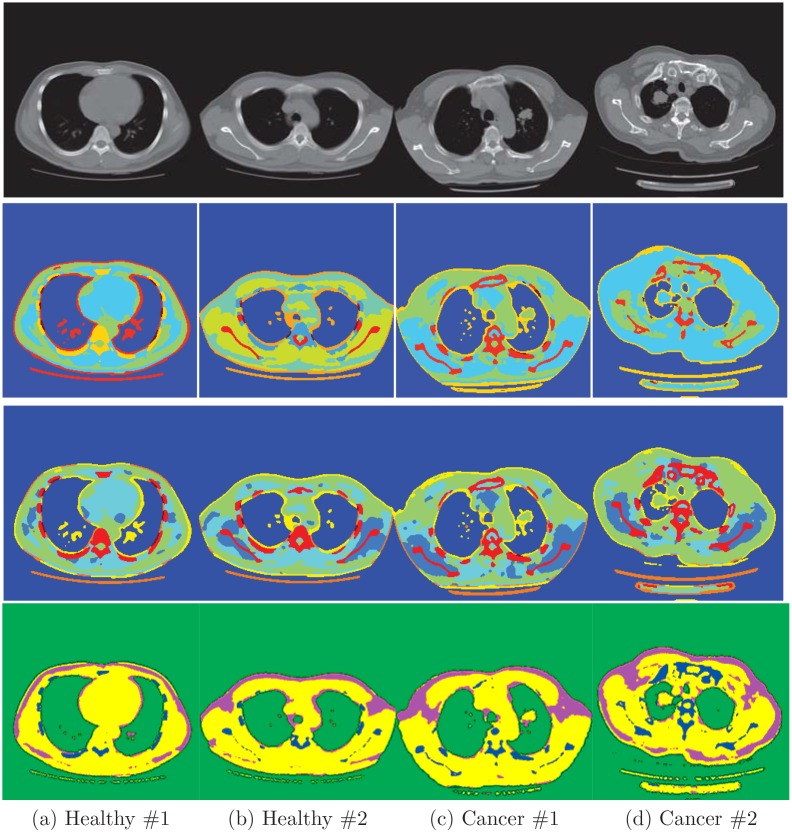
Segmentation results for four slices from four patient. The first row shows the original images; the second row is segmentation results by applying Dirichlet process (DP) model to each image individually; the third row is the results of the proposed hierarchical DP (HDP) model, the last row is the segmentation results of random walk.

In this section, we evaluate the proposed model to segment clinical lung CT images. We validate its accuracy by comparing its results with conventional Bayesian segmentation method based on Dirichlet process and the classical random walk algorithm. Specifically, we manually set the markers for the seeds initialization stage in random walk model. The clinical dataset are collected from 4 patients. Two of them are normal, the other two was undergoing lung cancer as indicated in Fig 1(c) and 1(d). All four acquisitions were performed with the approval of the Health Science Research Ethics Committee of Taizhou Municipal Hospital. The images were obtained on a GE lightSpeed VCT scanner (General Electric Company) at Taizhou Municipal Hospital. For each scan, a series of 5-mm thin slices that covered the lung region were acquired contiguously. Pixel size is 0.7031mm and the reconstructed image has size of 512 × 512. There is no additional pre-processing for all CT images after we obtained them from the scanner.

We conduct two groups of experiments to demonstrate the efficacy of segmentation algorithms. We first test the proposed model on four slices from the patient who has a tumor in the right lung. Then we select one representative slice from each patient and use the hierarchical model to segment all the four slices simultaneously to examine the effect of category sharing. Unlike many conventional models that are designed to segment one specific tissue or object, our model characterizes all objects within the image. To quantitatively test the accuracy, we have a professional physician manually delineate several objects including vertebral column, tumor, and lungs on the selected slices and use them as ground truth. Specifically, the physician manually traced the right and left lung borders, the vertebral and tumor. We compute the accuracy with Jaccard index (JI)

$$JI=\frac{A\cap B}{A\cup B}$$ (13)

The Jaccard index measures the overlap of two regions *A* and *B*, *A* being the manual tracing of an object, *B* the segmented area. JI being 1 means perfect overlap while 0 represents non-overlap.

### Segmentation within the same patient

**Fig 2:**
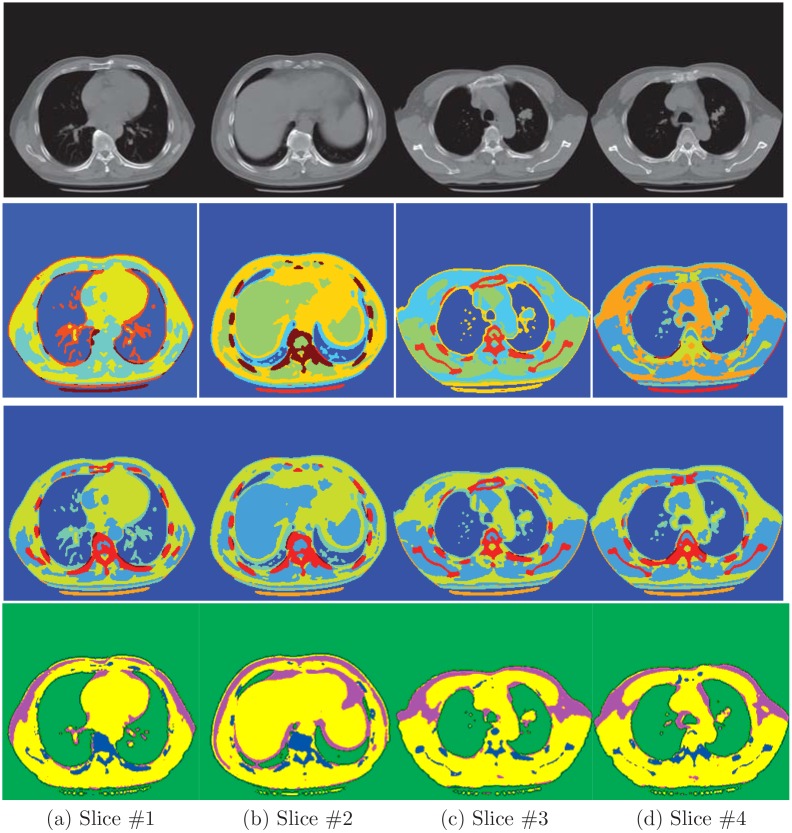
Segmentation results for four slices from the same patient. The first row shows the original images; the second row is segmentation results by applying Dirichlet process (DP) model to each image individually; the third row is the results of the proposed hierarchical DP (HDP) model, the last row is the segmentation results of random walk.

The first row of Fig 2 shows the four selected slices from the patient who was undergoing lung cancer. Due to the nonparametric nature of Dirichlet process, it infers the number of clusters from the data itself. As a result, it is prone to over-segmenting or less-segmenting from the limited information of input data, which is what happened in the first slice of the second row. We can see that it did not recognize the vertebral column and mis-segment them as fat tissue. But, instead, the hierarchical model that utilizes the information from other slices correctly identified the right object. Other than that, both models seem to give similar results. As for random walk, we set four kinds of seeds to start the diffusion process. We can see in the forth row of Fig 2 that random walk gives simpler segmentations as some organs are merged with others.

**Fig 3:**
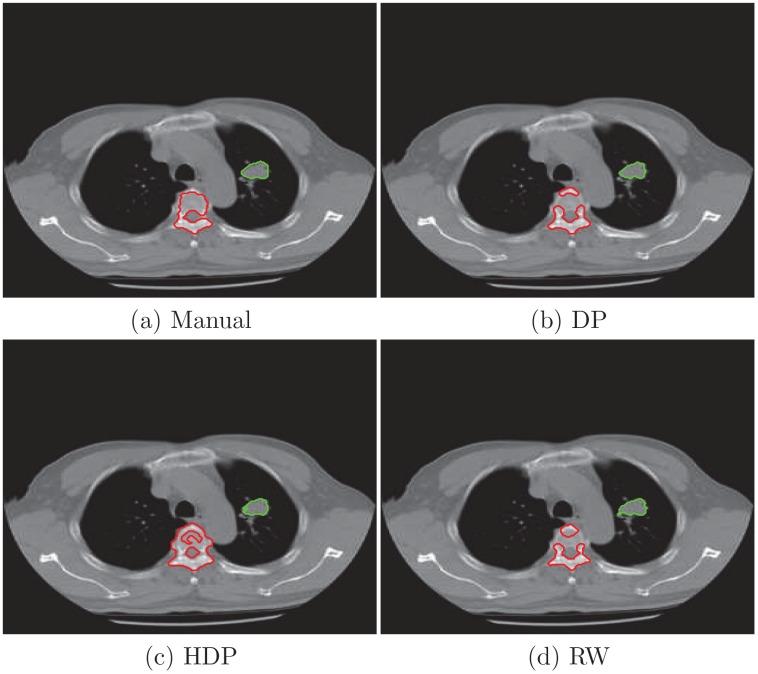
Object borders by manual tracing and estimates from segmentation algorithms for the third slice. (a) manual analysis from a physician; (b) automatic results given by DP model; (c) automatic result of HDP model; (d) automatic result of random walk.

**Fig 4:**
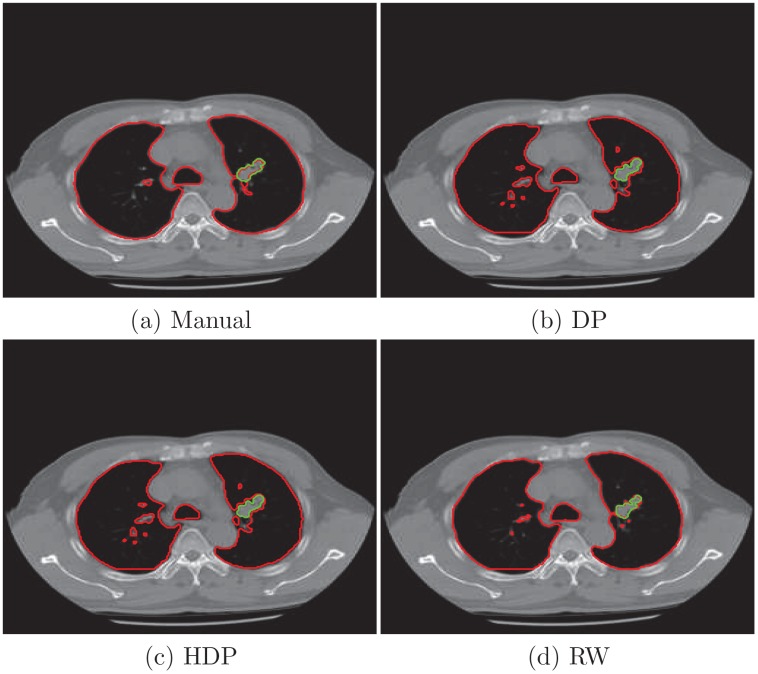
Object borders from manual tracing and segmentation algorithms for the forth slice. (a) manual analysis from a physician; (b) automatic results given by DP model; (c) automatic result of HDP model; (d) automatic result of random walk.

To closely examine the segmentation results, we plot the borders of two objects from the third and forth slice and compare them with the manual analysis. In Fig 3 we delineate vertebral column and the tumor in the third slice of the right lung. We also present the lung and tumor segmentation of the forth slice in Fig 4. Their Jaccard indexs in Table 1 show that the proposed algorithm more accurately extracts the objects in three cases. While the random walk model is slightly better for segmenting the tumor in the third slice.

**Table 1:** Jaccard index for segmented objects versus manual tracing. DP stands for segmentation model based on Dirichlet process, RW represents random walk model.

	Slice 3		Slice 4	
method	Vertebral column	tumor	lung	tumor
DP	0.42	0.84	0.80	0.80
RW	0.47	0.87	0.75	0.70
Ours	0.51	0.85	0.85	0.83

### Segmentation across patients

As we mentioned earlier, the motivation for the proposed hierarchical Bayesian model is the concept of category sharing in lung CT images, which is not limited to slices from the same patient. In this section, we extract one slice of CT scan near the pulmonary region from each of the four patients and segment them with various models so as to validate this very concept.

Fig 1 shows the segmentation results of the four slices. Same as the last section, single DP model and hierarchical model seem to give similar results. But looking closely at the details, we can find the difference. For instance, spine structures identified by single DP model in the second and forth patient are smaller than the estimates of the hierarchical model. Moreover, as we can see in Fig 1(c) and 1(d), it shows that the tumors are surrounded by a thin layer of fat. It is in accordance with clinical knowledge but can barely be found out by human eyes in the CT images. On the other hand, the random walk algorithm does not produce fine segmentation as the nonparametric models.

**Fig 5:**
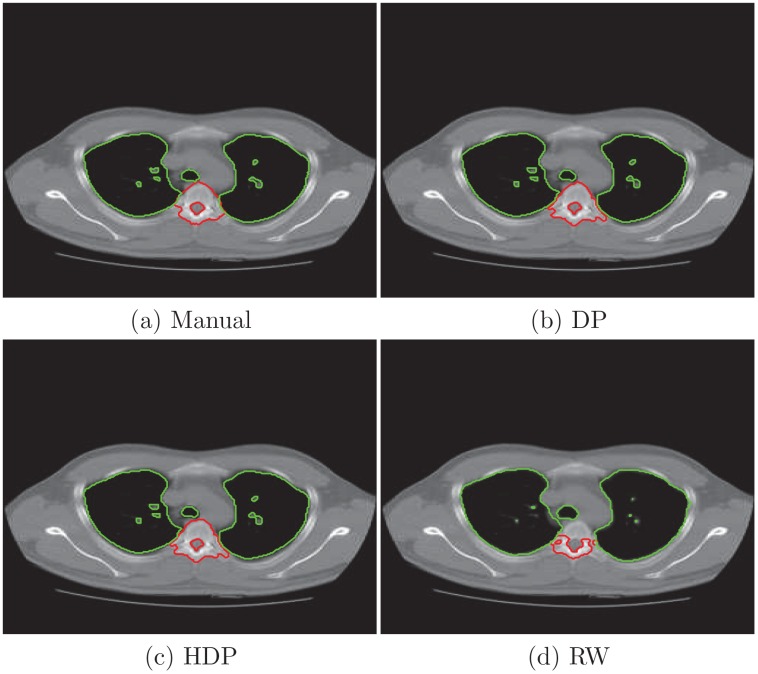
Segmentation results from slices from four patients. The first row shows the original images, the second row is segmentation results by applying DP model to each image individually, the third row is the results of the proposed model, the last row is the segmentation results of random walk.

**Fig 6:**
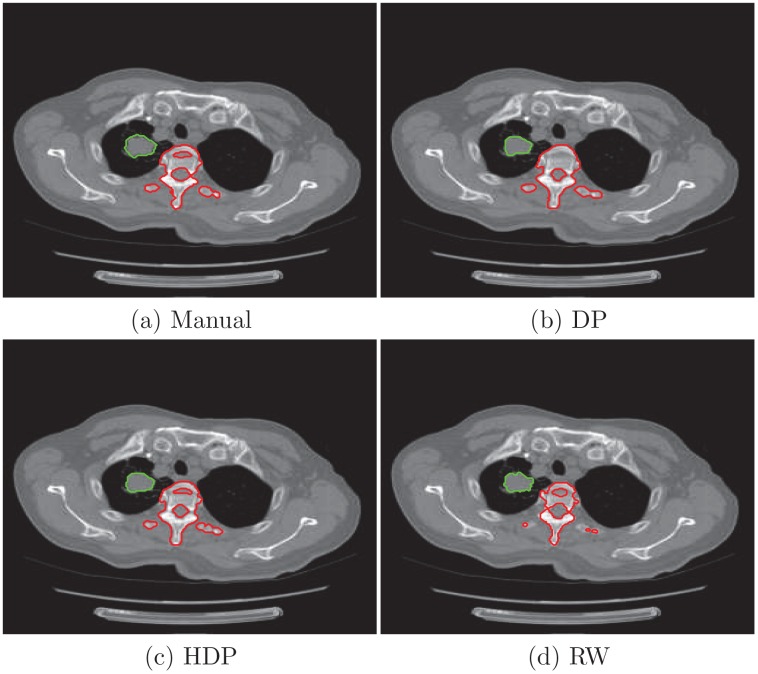
Segmentation results from slices from four patients. The first row shows the original images, the second row is segmentation results by applying DP model to each image individually, the third row is the results of the proposed model, the last row is the segmentation results of random walk.

We also present the borders of segmented objects from the second and forth patient. The physician manually traces the outlines of lung and vertebral column for the second patient (Fig 5), tumor and the vertebral column for the forth patient (Fig 6). From their Jaccard index in Table 2, we can see that the random walk algorithm performs poorly on the vertebral column for both two patients. While both single DP and HDP models give very accurate estimates of lung for the second patient. Overall the proposed hierarchical model achieves better accuracy over the examined objects.

**Table 2:** Jaccard index for segmented vertebra and lungs. DP stands for single segmentation model based on Dirichlet process, RW represents random walk model.

	Slice 2		Slice 4	
method	Vertebral column	lung	Vertebral column	tumor
DP	0.80	0.98	0.76	0.73
RW	0.29	0.90	0.64	0.79
Ours	0.83	0.97	0.81	0.79

## Discussion

We have developed a hierarchical Bayesian model to simultaneously segment multiple images and apply it to lung CT scans. Its main motivation is the concept of category sharing among CT slices that images from different patients or slices roughly share the same set of objects. We use the DP model to cluster the pixels, and we also encourage similarity between proximal points by incorporating MRF. We then formulated the hierarchical Dirichlet process model to enforce each DP model to share the same set of object category. We derived the inference algorithm for the proposed model based on Gibbs sampling to compute the posteriori for segmentation and use the mode as our final estimates. While this model is tested on lung CT data, it is generally applicable to a wide variety of clustering and segmentation problems in medical imaging.

In the experiment section, we collect lung CT images from the GE LightSpeed VCT scanner for both healthy and cancer patients to validate the segmentation algorithms. The conventional DP segmentation model tends to less-segmenting images because the ill-conditioning of this high dimension problem. As in the first section of segmenting images from the same patient, the single DP model overlooks the entire vertebral column. On the other hand, the random walk algorithm is purely based on intensity value proximity. Consequently, it faces serious challenges near the ambiguous boundaries. For instance, the vertebral content has similar intensity with surrounding tissue, which leads to the vague structure and boundary lines. As a result, the JI of vertebral column in the second group of experiments is 0.29 for random walk. Overall, by combining information from the whole population, the hierarchical model gives more accurate and consistent estimates of segmentation. As can be seen in Tables 1 and 2, the proposed algorithm achieves superior results for most of the cases compared to the other two models. It is also less prone to less or over-segmentation. The hyper-paramters could also affect the segmentation results. In specifics, there are two hyper-parameters in the presented HDP method, the concentration coefficient *α*_0_ in HDP and the smoothing parameter for modeling image pixels with Markov random field model. Throughout the experiments, we set the concentration parameter with 0.01 and the smoothing parameter with 0.001. Generally speaking, larger coefficient *α*_0_ tends to produce small amount of segments (i.e, less-segmentation) while smaller concentration parameter may lead to over-segmentation. On the other hand, large smoothing parameter may over-smooth the image thus the resulting segmentation will have less clusters. But it is our observation during experiments that the hierarchical method is less sensitive to hyper-parameter setting than the single DP model. W hile the random walk algorithm is very sensitive to predefined number of segments.

As we mentioned before, many clinical routines for pulmonary disease require image segmentation as an introductory step. Computer based diagnosis (CAD) and image based radiotherapy also heavily rely on the accuracy of anatomy characterization for the following detection, localization, classification tasks. Conventional techniques based on contrast difference and predefined settings usually fail to characterize the pathological organs. Because most of the pathological structures are patient-specific and may not be observed before. Especially for lung CT scans, they often contain significant amount of pathologies. Because of the nonparametric nature of DP model, our method automatically discovers the entire category that can describe the components in each image. The enforcement of category sharing serves as our effort for regularization so that more robust and consistent estimates could be obtained across the whole dataset. As demonstrated in the experiments, our model gives more accurate estimates of the tumor in most cases.

Due to the limited time, we have only validated the model over four patients from real scanner. But we are currently collecting more data and aim to conduct experiments on massive datasets. Because of the increased inference space of this hierarchical model, current implementation requires much more intensive computations as well as longer inference time as shown in Table 3. In the future, we will explore more efficient solutions including stochastic inference, parallel computation based graphical processing unit (GPU) and variational inference methods.

**Table 3:** Inference time for presented three methods for segmenting four slices in Section. segmentation from the same patient. The computation time for DP and random walk is the combined time for segmenting four slices.

method	DP	HDP	random walk
	1 (min)	5 (min)	56 (s)
